# Drug Discovery in Spinal Cord Injury With Ankylosing Spondylitis Identified by Text Mining and Biomedical Databases

**DOI:** 10.3389/fgene.2022.799970

**Published:** 2022-02-25

**Authors:** Chenfeng Wang, Hongdao Ma, Weiqing Wu, Xuhua Lu

**Affiliations:** Department of Orthopaedics, Shanghai Changzheng Hospital, Naval Medical University, Shanghai, China

**Keywords:** spinal cord injury, ankylosing spondylitis, text mining, drug discovery, bioinformatic analysis

## Abstract

Spinal cord injury (SCI) and ankylosing spondylitis (AS) are common inflammatory diseases in spine surgery. However, it is a project where the relationship between the two diseases is ambiguous and the efficiency of drug discovery is limited. Therefore, the study aimed to investigate new drug therapies for SCI and AS. First, text mining was used to obtain the interacting genes related to SCI and AS, and then, the functional analysis was conducted. Protein–protein interaction (PPI) networks were constructed by STRING online and Cytoscape software to identify hub genes. Last, hub genes and potential drugs were performed after undergoing drug–gene interaction analysis, and MicroRNA and transcription factors regulatory networks were also analyzed. Two hundred five genes common to “SCI” and “AS” identified by text mining were enriched in inflammatory responses. PPI network analysis showed that 30 genes constructed two significant modules. Ultimately, nine (*SST*, *VWF*, *IL1B*, *IL6*, *CXCR4*, *VEGFA*, *SERPINE1*, *FN1*, and *PROS1*) out of 30 genes could be targetable by a total of 13 drugs. In conclusion, the novel core genes contribute to a novel insight for latent functional mechanisms and present potential prognostic indicators and therapeutic targets in SCI and AS.

## Introduction

Spinal cord injury (SCI) is a serious complication of traumatic diseases such as spinal fracture or dislocation, burdening families, economics, and society (GBD 2017 US Neurological Disorders Collaborators et al., 2021). On the other hand, ankylosing spondylitis (AS) is a chronic disease that mainly affects the spine and accumulates the sacroiliac and surrounding joints (Braun and Sieper, 2007). However, the relationship between the two diseases is unclear and the efficiency of drug discovery is limited. In addition, if patients with AS happen to have SCI, then the question whether AS aggregates the injury degree of the spinal cord needs to be answered. Therefore, mining the underlying pathomechanism is a necessary way to further understand the relationship between the two diseases and discover potential therapies for destroying their interaction.

There is possibly an inevitable connection between the SCI and AS. The incidence of SCI appears to be higher in patients with AS than in the general population, which, of course, is directly related to the increasing incidence of vertebral column fracture in patients with AS (Jacobs and Fehlings, 2008). Under the pathologic mechanism of the disease, combined with an unstable spine, the AS population is predisposed to highly distracting injuries and spinal epidural hematoma formation further increases the severity of SCI. Hence, they likely share similar pathogenesis, genes, and antigens.

In recent years, with the development of bioinformatics methods and computational technology, the genetic mechanism behind diseases can be interpreted to investigate deleterious variants associated with target drugs, such as cancers, degenerative diseases, and COVID-19 (Cheng et al., 2016; Woolston et al., 2019; DiNardo et al., 2020; Li et al., 2021). Having been applied in exploring biomarkers, searching for drug targets, and studying molecular regulation, bioinformatics analysis acts as a catalyst to clarify the potential pathogenic agents (Li et al., 2019a; Tolios et al., 2020). Text mining of biomedical literature has been understood as a valid way to reveal the relationship between genes and pathologies while combining with other bioinformatical tools is an effective protocol to verify the existing therapy.

**FIGURE 1:**
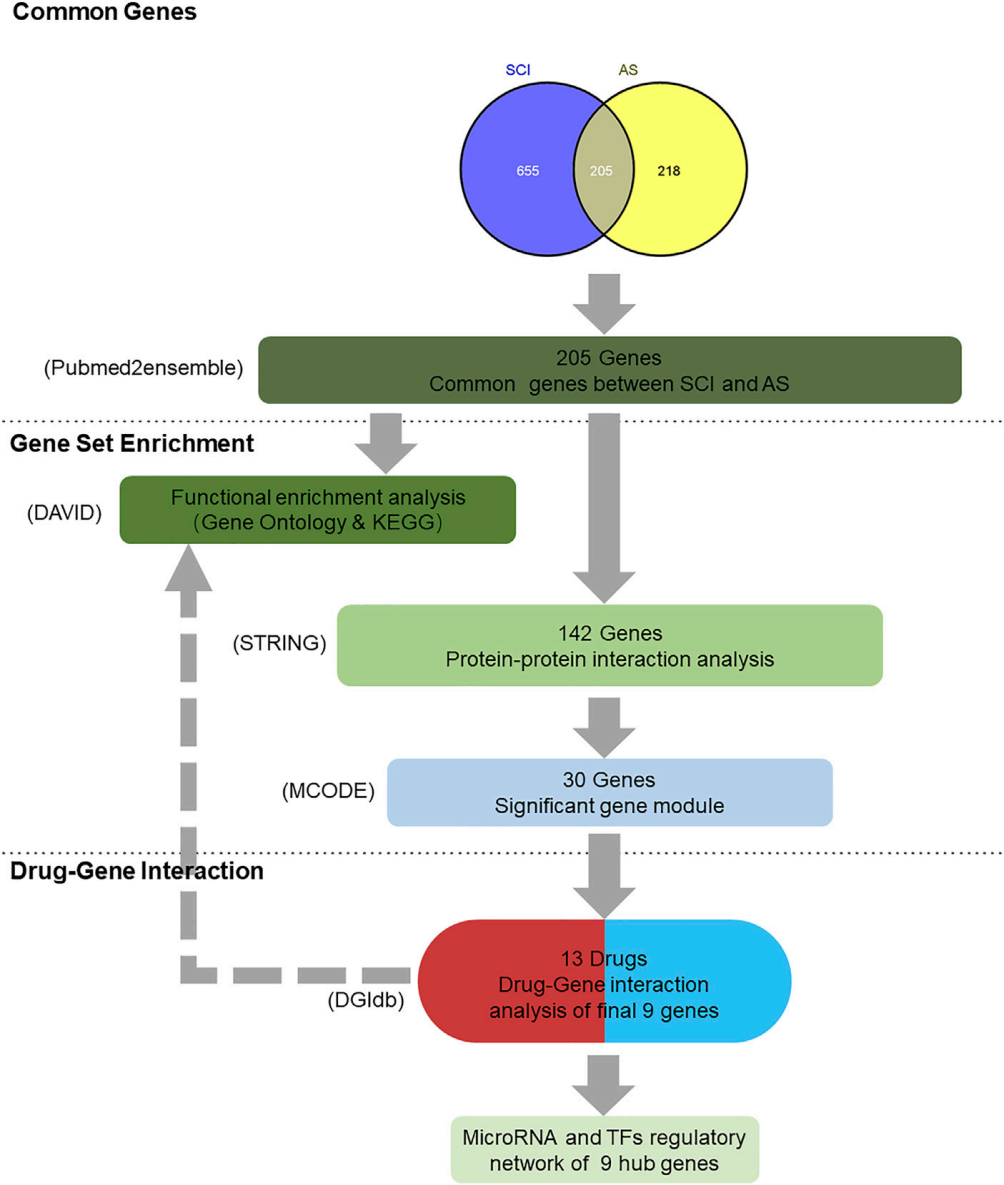
Summary of overall data mining result. 1) Text mining: 860 genes were found by using the searching term “spinal cord injury” and 423 genes were found *via* the term “ankylosing spondylitis” in pubmed2ensemble, ultimately interacting 205 genes. 2) Gene set enrichment: DAVID functional enrichment analysis was performed using biological process, cellular component, molecular function, and signaling pathways analysis. Next, 30 genes were screened out by using the STRING and Cytoscape software. 3) Drug–gene interaction and functional analysis; 30 genes were imported into the DGIdb and 13 drugs were regarded as the potential medical therapy, whereas nine genes were selected as the final genes that completed the functional analysis. 4) Nine hub genes were participating in the analysis of their MicroRNA and TFs regulatory network.

Despite extensive research on SCI and AS, there still is a gap in understanding intersecting genes and latent targets for the treatment of SCI and AS. This study aimed to mine the correlations between the SCI and AS, further obtaining new drugs to control the progression of the two, including text mining, genetic functional analysis, protein–protein interaction (PPI) analysis, drug–gene interaction, and related regulatory molecular analysis. First, common genes were obtained from the SCI and AS preliminary gene lists. Second, interacting genes conducted functional analysis for clarifying related functions and pathways. Third, PPI analysis is to further screen out core genes and clusters, further purposing drug selection. Last, the hub genes obtained from drug–gene interaction were undergone MicroRNA analysis and transcriptional factors (TFs) regulatory network to explore the potential mechanisms (Figure 1).

## Methods and Materials

### Text Mining

Pubmed2ensemble, an available website, could be utilized to conduct text mining (http://pubmed2ensembl.ls.manchester.ac.uk/). Relevant kinds of literature and databases are mined in pubmed2ensemble to obtain significant genes. In this study, two queries were performed with “spinal cord injury” and “ankylosing spondylitis”. Two gene lists were generated, and each gene hits were extracted to acquire the common genes for the next analysis.

### Gene Ontology and Signal Pathways Analysis

The DAVID (https://david.ncifcrf.gov/) was used to conduct gene ontology (GO) and signal pathways annotations for the genes corresponding to the SCI and AS intersection. Biological process, cellular component, and molecular function genes participated in explained functional information while performing pathways analysis with the Kyoto Encyclopedia of Genes and Genomes (KEGG). False discovery rate (FDR) < 0.05 was set as the cutoff.

### Protein–Protein Interaction Analysis and Gene Module Analysis

First, the STRING database (https://www.string-db.org/), a useful tool to complete functional protein association networks, was selected to analyze the PPI. Second, we uploaded the genes to the site, and the maximum interaction score >0.9 (high confidence) was regarded as an important screening indicator. Third, the TSV file downloaded from the STRING was imported into the Cytoscape software for the Molecular Complex Detection (MCODE), finally building up the gene modules (cluster) to own the hub genes, which were applied to the subsequent steps of drug–gene interactions.

### Drug–Gene Interaction and Functional Analysis

It was realizable for us to explore the interaction between drugs and genes, which would help us to search for the potential therapy target of SCI and AS. The drug–gene interaction database (https://www.dgidb.org/) is the same as the abovementioned website, open to everyone eager to find effective medicines to treat SCI and AS. The module genes would match with existing drugs whose criteria were obvious interaction type and approved by countries. Besides, final genes after interacting were continued to take part in the process of functional analysis, and FDR of consequences <0.05 was recognized as statistically significant.

### MicroRNA Analysis and TFs Regulatory Network of Hub Genes

Targetscan (http://www.targetscan.org/) could be utilized to search for the microRNA of hub genes. In addition, TFs of core genes were relying on another online database named NetworkAnalyst (https://www.networkanalyst.ca/), a comprehensive network visual analytics platform for gene expression analysis.

## Result

### Identification of Common Genes

After eliminating the duplication of gene symbols, 605 genes were related to SCI, 218 genes were related to AS, and 205 common genes were found from text mining with conceptions of “spinal cord injury” and “ankylosing spondylitis”.

### Gene Ontology and Signal Pathways Analysis

Common 205 genes were participating in the process of GO and KEGG analysis. Consequently, the number of GO terms for the biological process is 1,750, that of the cellular component is 37, and that of molecular function is 31. The five most enriched annotations of the biological process were 1) “response to organic substance” (FDR = 2.85E-35), 2) “response to external stimulus” (FDR = 7.78E-32), 3) “positive regulation of multicellular organismal process” (FDR = 1.62E-31), 4) “inflammatory response” (FDR = 1.81E-31), and 5) “positive regulation of response to stimulus” (FDR = 1.70E-30), respectively, containing 115, 96, 51, 58, and 93 genes from the query set. As for cellular component, the five most enriched annotation were 1) “extracellular space” (FDR = 1.71E-32), 2) “extracellular region part” (FDR = 2.19E-25), 3) “extracellular region” (FDR = 1.07E-24), 4) “cell surface” (FDR = 1.25E-22), and 5) “external side of plasma membrane” (FDR = 6.27E-19), containing 85, 123, 133, 54, and 31 genes. There are also five most enriched annotation of molecular function, 1) receptor binding (FDR = 4.41E-28), 2) cytokine receptor binding (FDR = 1.27E-18), 3) cytokine activity (FDR = 5.97E-17), 4) growth factor activity (FDR = 1.98E-12), and 5) identical protein binding (FDR = 3.12E-8).

**FIGURE 2:**
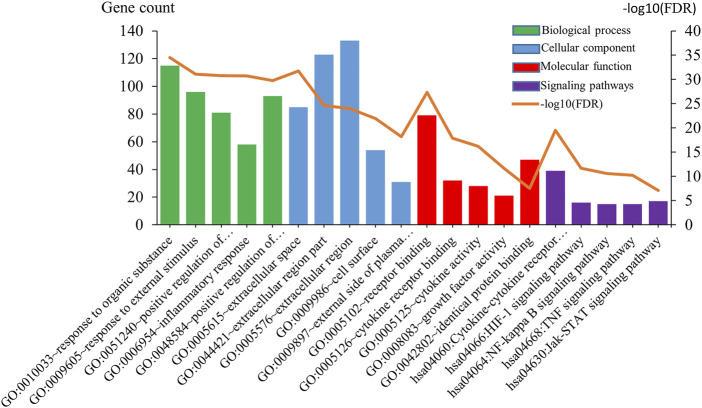
Gene ontology and signal pathways analysis. Gene ontology analysis classified common genes into biological processes, cellular components, and molecular functions. Green bar charts represent the biological process, blue bar charts represent the cellular component, red bar charts represent the molecular function, purple bar charts represent the signaling pathways, and orange line chart represents −log10 (FDR).

In addition, the most enriched annotations of signaling pathways were that 1) Cytokine-cytokine receptor interaction (FDR = 3.17E-20), 2) HIF-1 signaling pathway (FDR = 2.22E-12), 3) NF-kappa B signaling pathway (FDR = 2.46E-11), 4) TNF signaling pathway (FDR = 5.86E-11), 5) Jak-STAT signaling pathway (FDR = 7.66E-8), respectively containing 39, 16, 15, 15, and 17 genes from the query set (Figure 2).

### Module Screening of Protein–Protein Interaction Network

**FIGURE 3:**
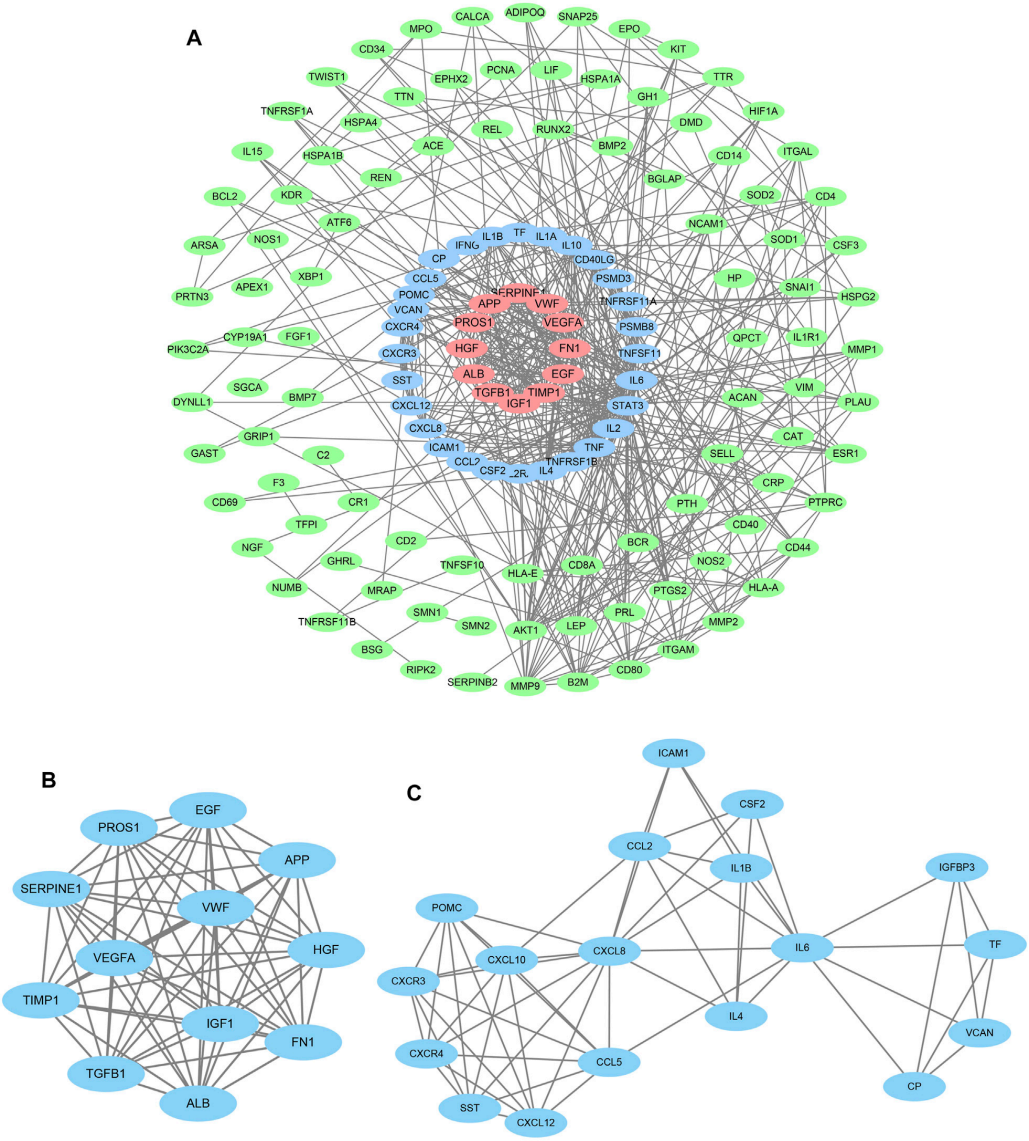
Protein–protein interaction analysis and gene module analysis. **(A)** Based on the STRING online database, 142 genes and 507 edges formed the network which was under the maximum interaction score >0.9 (high confidence). **(B)** Cluster 1: the first significant module from the PPI network, containing 12 nodes and 86 edges. **(C)** Cluster 2: the second significant module from the PPI network, containing 18 nodes and 54 edges.

Meeting the limitation of high confidence and hiding non-interacting genes, candidate genes were selected to construct a PPI network *via* multiple protein functions of the STRING database. A “tsv” file was exported from the site and then imported into the Cytoscape software for the clustering analysis, illustrating that there are 142 nodes and 507 edges (Figure 3A). MCODE app, a necessary tool, was used to complete cluster analysis to further screening for the target genes, eventually acquiring various clusters. Cluster 1 constructed by 12 nodes and 86 edges and cluster 2 built up by 18 nodes and 58 edges were chosen to collect important genes (Figures 3B,C).

### Potential Therapeutics and Functional Analysis

**FIGURE 4:**
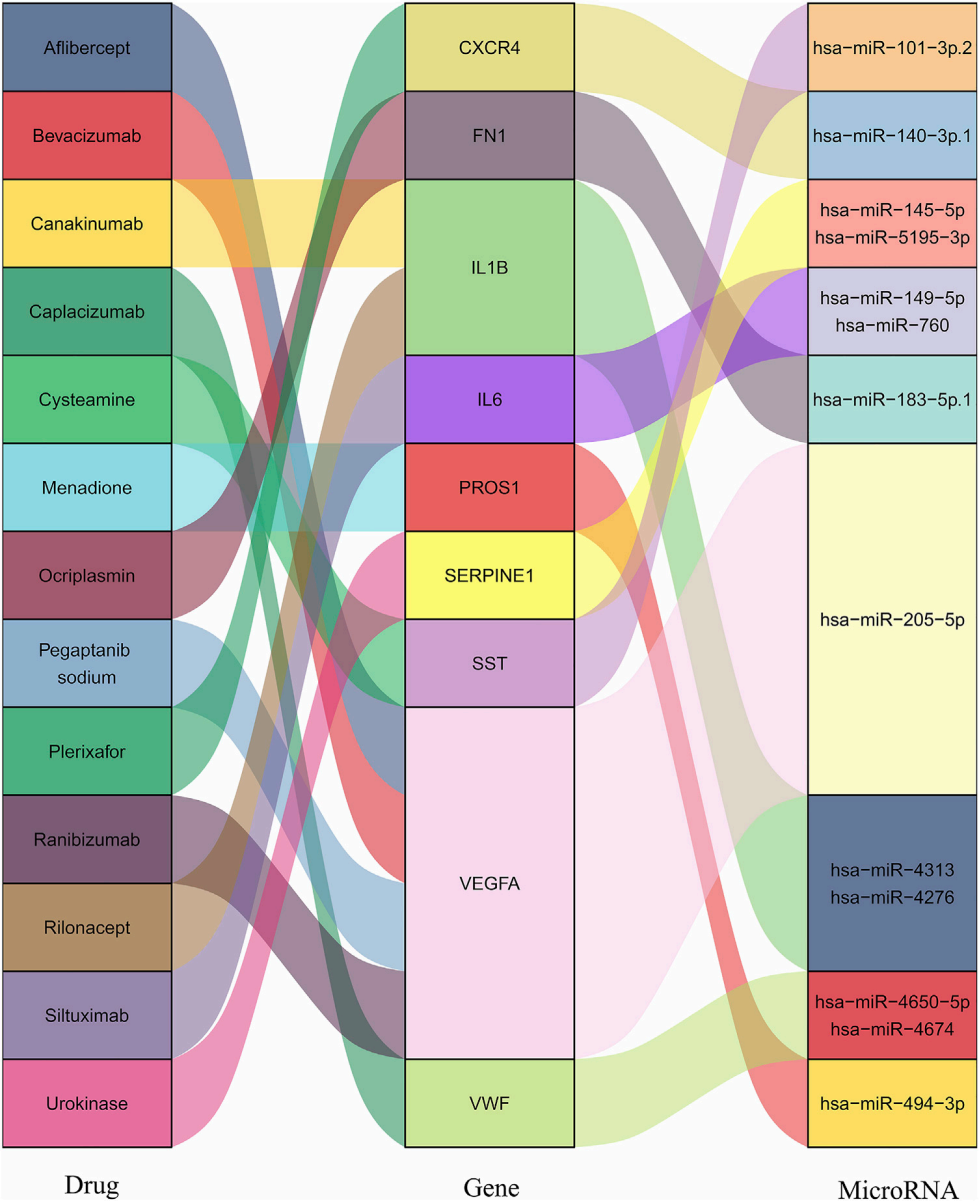
The interactions of hub genes with drugs and MicroRNA. The interaction of nine hub genes with 13 drugs and MicroRNA.

Drug–gene interaction is the last step to search for the potential signatures and target medicines, investigating existing drugs to alleviate the damaging degree of SCI and AS. Among the PPI and MCODE analysis, a hub gene list that had been eliminated the duplicating part was a channel to the DGIdb (http://www.dgidb.org/), which was consist of 27 various clinical databases and literature, such as DrugBank and NCBI PubMed. With the limitations of presence or absence of approvals and definitive drug–gene interacting type, a sum of 13 drugs was shown in Table 1 and Figure 4, and the interacting score of Cysteamine was highest. Depending on the target drugs analysis result, nine genes (*SST*, *VWF*, *IL1B*, *IL6*, *CXCR4*, *VEGFA*, *SERPINE1*, *FN1*, and *PROS1*), which acted as the core to take part in the occurrence and development of SCI and AS underwent functional analysis (Table 2), which clarified HIF-1 signaling pathway and PI3K-Akt signaling pathway played an important role in the progression of the two diseases.

**TABLE 1:** Potential drugs targeting genes with SCI and AS association.

Number	Drug	Gene	Type	Score^a^	Approved?	PMID
1	Aflibercept	VEGFA	Inhibitor, Binder	2.35	Yes	22813448
2	Bevacizumab	VEGFA	Inhibitor	1.02	Yes	18182667
3	Canakinumab	IL1B	Inhibitor, Binder	10.34	Yes	19169963
4	Caplacizumab	VWF	Inhibitor	13.67	Yes	None
5	Cysteamine	SST	Binder	18.23	Yes	2653642
6	Menadione	PROS1	Activator	1.14	Yes	12033454
7	Ocriplasmin	FN1	Inhibitor	1.29	Yes	23193358
8	Pegaptanib sodium	VEGFA	Antagonist	3.36	Yes	23953100
9	Plerixafor	CXCR4	Antagonist, Agonist	8.93	Yes	17715128
10	Ranibizumab	VEGFA	Inhibitor	8.81	Yes	18046235
11	Rilonacept	IL1B	Inhibitor, Binder	3.45	Yes	23319019
12	Siltuximab	IL6	Antagonist	10.21	Yes	8823310
13	Urokinase	SERPINE1	Inducer, Substrate	3.19	Yes	12709915

Each drug–gene interaction ensured that the hypothetical drug had an expected effect on the condition. The link to the source was tracked to confirm the report and evaluate related metadata such as approval status and available route drug use. Drugs that targeted the candidate genes through appropriate interactions were collected in the final list.

a The score is the combined number of database sources and PubMed references.

**TABLE 2:** Summary of gene set enrichment analysis.

Category	Term	Count	FDR^a^	Genes
Biological process	Leukocyte migration	7	4.83E-06	IL6, IL1B, PROS1, SERPINE1, FN1, CXCR4, VEGFA
Biological process	Platelet degranulation	5	5.35E-05	VWF, PROS1, SERPINE1, FN1, VEGFA
Biological process	Cell motility	8	5.35E-05	IL6, SST, IL1B, PROS1, SERPINE1, FN1, CXCR4, VEGFA
Biological process	Localization of cell	8	5.35E-05	IL6, SST, IL1B, PROS1, SERPINE1, FN1, CXCR4, VEGFA
Biological process	Locomotion	8	1.13E-04	IL6, SST, IL1B, PROS1, SERPINE1, FN1, CXCR4, VEGFA
Cellular component	Platelet alpha granule lumen	5	5.73E-07	VWF, PROS1, SERPINE1, FN1, VEGFA
Cellular component	Platelet alpha granule	5	1.02E-06	VWF, PROS1, SERPINE1, FN1, VEGFA
Cellular component	Secretory granule lumen	5	1.18E-06	VWF, PROS1, SERPINE1, FN1, VEGFA
Cellular component	Cytoplasmic membrane-bounded vesicle lumen	5	1.65E-06	VWF, PROS1, SERPINE1, FN1, VEGFA
Cellular component	Vesicle lumen	5	1.65E-06	VWF, PROS1, SERPINE1, FN1, VEGFA
Molecular function	Receptor binding	7	0.001232431	IL6, VWF, SST, IL1B, SERPINE1, FN1, VEGFA
Molecular function	Protease binding	3	0.045365023	VWF, SERPINE1, FN1
Molecular function	Growth factor receptor binding	3	0.045365023	IL6, IL1B, VEGFA
KEGG pathway	Complement and coagulation cascades	3	0.046494339	VWF, PROS1, SERPINE1
KEGG pathway	HIF-1 signaling pathway	3	0.046494339	IL6, SERPINE1, VEGFA
KEGG pathway	PI3K-Akt signaling pathway	4	0.046494339	IL6, VWF, FN1, VEGFA

With a strict level, a *p*-value cutoff was set. Among the most importantly enriched biological process, cellular component, molecular function, and KEGG pathways above the cutoff, those most relevant to SCI and AS pathology were chosen from the researches and literature.

a FDR correction was performed to control for the false positive.

### MicroRNA and TF Regulatory Network

**FIGURE 5:**
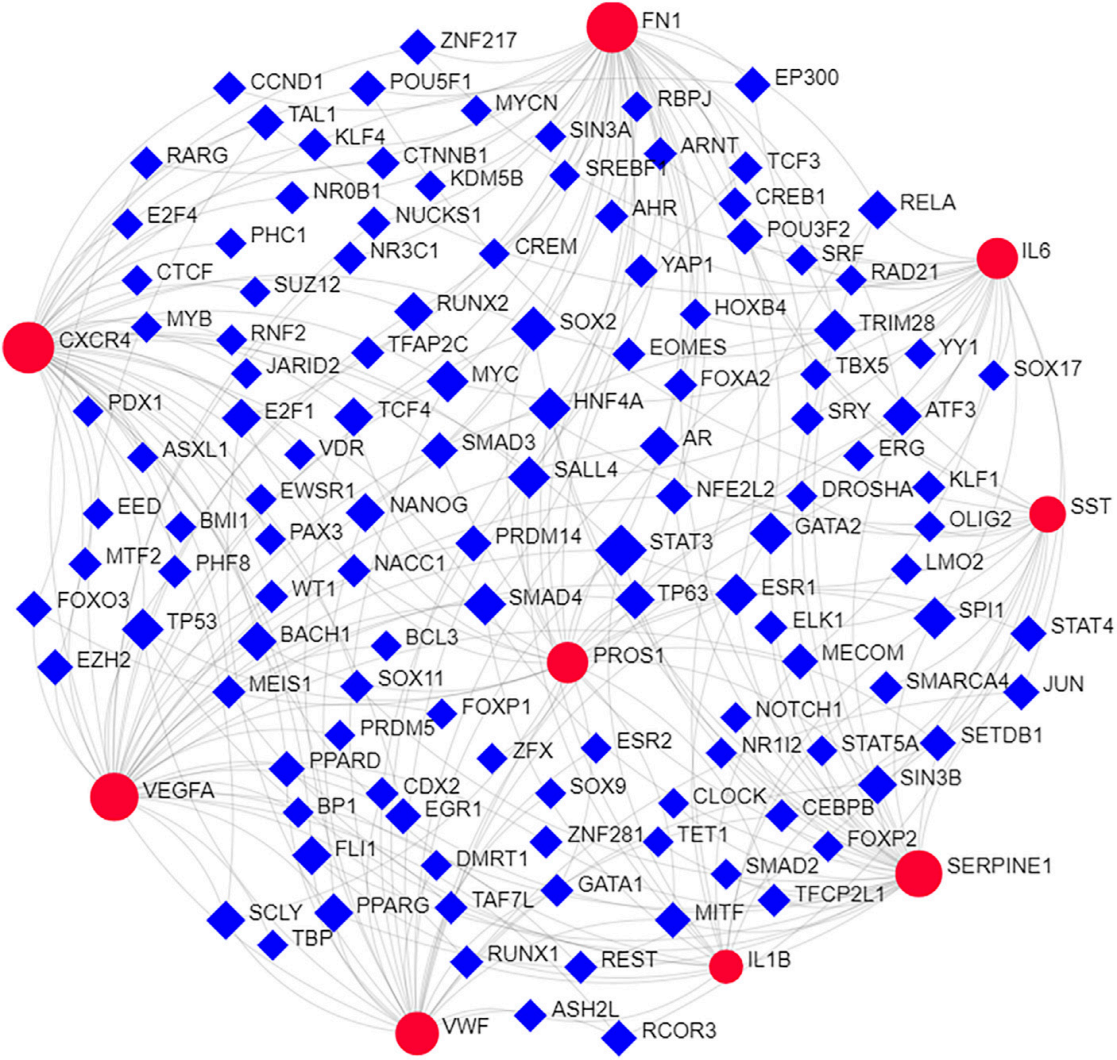
The interactions of hub genes with TFs. The TFs regulatory network of nine hub genes. Red nodes represented nine hub genes and blue squares represented TFs.

Nine hub genes were corresponding to one or two conserved MircoRNA shown in Figure 4. The TFs regulatory network consisted of nine genes and 122 TFs (Figure 5). *CXCR4* was regulated by 47 TFs, *FN1* was regulated by 46 TFs, *VEGFA* was found to be regulated by 39 TFs, and *SERPINE1* was regulated by 36 TFs. The numbers of *VWF*, *IL6*, *PROS1*, *SST*, and *IL1B* were 29, 25, 25, 18, and 15, respectively. In addition, 11 TFs were found with an interacting degree >4 in the network, implying they may play a key role in the progress of diseases (Table 3).

**TABLE 3:** Summary of potential TFs of hub genes.

TFs	Genes	Count
STAT3	SST, VWF, IL1B, IL6, CXCR4, VEGFA, SERPINE1, FN1, PROS1	9
SOX2	IL6, CXCR4, VEGFA, SERPINE1, FN1, PROS1	6
MYC	VWF, IL6, CXCR4, VEGFA, FN1	5
SMAD4	IL1B, CXCR4, VEGFA, SERPINE1, FN1	5
TRIM28	IL6, VEGFA, SERPINE1, FN1, PROS1	5
SALL4	IL1B, IL6, CXCR4, FN1, PROS1	5
GATA2	VWF, IL1B, IL6, SERPINE1, FN1	5
SPI1	SST, IL1B, IL6, SERPINE1, PROS1	5
ESR1	VWF, VEGFA, SERPINE1, FN1, PROS1	5
HNF4A	VWF, IL1B, VEGFA, SERPINE1, FN1	5
TP53	VWF, IL1B, CXCR4, VEGFA, PROS1	5

## Discussion

Leading to lifelong disability, SCI, a devastating disease, is damage to the spinal cord. As for AS, the autoimmune disease is characterized by inflammation of the sacroiliac joints and the attachment points of the spine. According to the study outcomes, patients with AS are more likely to fracture and develop SCI. However, there has been little or no research into drug discovery for SCI and AS diseases treatment. Therefore, we designed this study to identify the potential drugs of therapeutic value, further providing references for clinical diagnosis and treatment. These 13 drugs target the following nine genes: *VEGFA* (four drugs), *IL1B* (two drugs), *SST*, *VWF*, *IL6*, *CXCR4*, *SERPINE1*, *FN1*, and *PROS1* (one drug each).

*CXCR4* (C-X-C motif chemokine receptor 4), a member of the GPCR family, and *CXCL12* constitute an axis to modulate the nerve system. The expression of the *CXCL12/CXCR4* axis was increasing in the patients with SCI and AS (Knerlich-Lukoschus et al., 2011; Tysseling et al., 2011). Besides, a study found that *CXCR4* could accelerate the ossification of fibroblasts in patients with AS (He et al., 2019). *FN1* (fibronectin 1) is involved in the process of cell adhesion and migration (Cai et al., 2018) and could rely on the TGF-β/PI3K/Akt pathway to promote chondrocyte differentiation and collagen production in fractured bones (Zhang et al., 2021). As for *PROS1* (protein s), one of its roles is to inhibit tumor metastasis by mediating inflammation and immunization (Maimon et al., 2021). Downregulated *SERPINE1* (serpin family E member 1) could accelerate the progression of inflammation (Yang et al., 2017).

When it comes to the *VWF* (von Willebrand factor) and *VEGFA* (vascular endothelial growth factor), some reports have revealed the relationship between the two genes and the two diseases (Widenfalk et al., 2003; Divecha et al., 2005; Nesic et al., 2010; Taylan et al., 2012). Experiencing compressive injury, the levels of *VWF* and *VEGFA* expression in the rat spinal cord were decreased. Interestingly, the levels of two gene expressions were dramatically increased after decompression (Cheng et al., 2021), which was consensus with another study (Shen et al., 2020). Furthermore, *VWF* production in men with AS was more than normal people, and it is likely to be recognized as a biomarker of AS (Taylan et al., 2012). In addition, a study found that early *VEGF* delivery for SCI treatment attributed to its power of protecting vessels and inhibiting cell apoptosis was advantageous (Widenfalk et al., 2003), and it even serves as an important criterion for assessing SCI (Nesic et al., 2010).

GO analysis and KEGG enrichment analysis of DEGs showed that most DEGs were closely related to inflammatory response, response to external stimulus and response to organic substance. PI3k/Akt and *HIF-1* are the major signaling pathways that have been identified to be significant in inflammatory diseases. PI3K/Akt signaling pathway is inhibited by endoplasmic reticulum stress after SCI, which is activated to realize motor recovery and nerve protection (Li et al., 2019b). In addition, owning the function of regulating spinal cord regeneration, PI3K/Akt is regarded as a potential target for SCI treatment (Lu et al., 2018). Concerning the *HIF-1* signaling pathway, if combined with *VEGF*, then it is activated, secondary SCI will be inhibited, and local hypoxic ischemia environment will be improving, meanwhile promoting neurological recovery (Chen et al., 2017). Furthermore, the *HIF-1* signaling pathway that can also be explored to prevent SCI involves the apoptosis of neuronal cells (Wang et al., 2016). On the basis of the previous studies, *HIF-1* activity relies on the *HIF-1α* that is particularly controlled by the PI3K/Akt signaling pathway at the post-transcriptional protein level (DeBerardinis et al., 2008; Zhang et al., 2018).

The top five on the drug–gene interacting scoring system were Cysteamine (*SST* binder), Caplacizumab (*VWF* inhibitor), Canakinumab (*IL1B* inhibitor), Siltuximab (*IL6* antagonist), and Plerixafor (*CXCR4* inhibitor). Owning the highest interacting score in these drugs, Cysteamine deserves to be more explored for its therapeutic value for SCI patients with AS, which is mainly applied to cure Cystinosis (Hollywood et al., 2020), atherosclerosis (Wen et al., 2019), and Parkinson’s disease (Cicchetti et al., 2019). Caplacizumab is only used for acquired thrombotic thrombocytopenic purpura (Scully and Spero, 2019), whereas Siltuximab is mostly utilized for Castleman’s disease (van Rhee et al., 2014). Besides, Canakinumab has entered the clinical stage of lung cancer treatment, perhaps becoming the candidate for SCI or AS (Wong et al., 2020). On the basis of the existing researches, inhibition of *CXCR4* could alleviate inflammatory pain induced by ischemia–reperfusion (Li et al., 2016).

More than a quarter of identified drugs target *VEGFA*, including Aflibercept, Bevacizumab, Pegaptanib sodium, and Ranibizumab. Bevacizumab approved by the US Food and Drug Administration (FDA) has been used in the management of neurosurgery diseases such as radiation-induced myelopathy and gliomas (Psimaras et al., 2016; Levenbaum et al., 2019). Surprisingly, some drugs (Aflibercept, Pegaptanib sodium, and Ranibizumab) for the therapy of age-related macular degeneration may also be a benefit for SCI patients with AS. As mentioned before, *VEGFA* owns the function of inducing angiogenesis, indicating that it plays a key role in the SCI and AS. Although the above drugs are not currently used for AS and SCI, they are approved by the FDA and may be possibly approved in the therapy of the two diseases in the future.

Interestingly, it is worth noting that *STAT3* (signal transducer and activator of transcription 3), as the most connected TF, and MicroRNAs, respectively, corresponding to final genes play a significant role in the development of AS and SCI. The expression of *STAT3* is high in patients with AS as well as SCI patients, so AS can further aggregate the degree of SCI and induce more severe inflammatory storms, finally influencing the prognosis of patients (Jo et al., 2020). Meantime, TFs and MicorRNA can also regulate the hub genes to mediate inflammations (Qiu et al., 2021). For example, MicroRNA-149 could suppress hepatic inflammatory response through antagonizing *STAT3* signaling pathway (Zhang et al., 2017). Besides, MicroRNA-183 regulates LPS-induced oxidative stress by targeting *FN1* (Xie et al., 2020) and MicroRNA-140-3p can ameliorate the progression of osteoarthritis *via* targeting *CXCR4* (Ren et al., 2020). Therefore, MicorRNA and TFs may act as important targets for the treatment of SCI patients with AS, providing direction for later researches.

All consequences suggested that common genes are not only responsible for the SCI and AS but also cover a wide range of digestive system neoplasm and blood system *via* cytokine-cytokine interaction, PI3K/Akt signaling pathway, and *HIF-1* signaling pathway. What calls for special attention is that those alternating genes can aggravate the severity of the SCI and AS, thus building up a vicious circle, and there is also a potential risk of tumor formation. Therefore, it is important to give early interventions for SCI patients with AS through potential drugs therapy. On the one hand, the progression of SCI and AS can be postponed by early treatment, finally breaking the vicious circle. On the other hand, tumors will not be given a chance to develop if intervention is used in the early phase, ultimately maintaining body physical activity to promote recovery.

Regrettably, the limitation of this study is that, with the update of the database we used, our study needs to catch up with the development to repeat the study. In addition, the criteria of screening out core genes are subjective, implicating objective criteria need to be constructed to identify the candidate.

## Conclusion

In conclusion, our study obtained 205 genes common in both SCI and AS. GO and KEGG analyses of intersecting genes might reveal a novel prospective relationship between SCI and AS. Consequently, the final nine genes (*SST*, *VWF*, *IL1B*, *IL6*, *CXCR4*, *VEGFA*, *SERPINE1*, *FN1*, and *PROS1*) and 13 drugs may provide therapeutic value for SCI patients with AS.

## Data Availability

The original contributions presented in the study are included in the article/Supplementary Material. Further inquiries can be directed to the corresponding author.
